# Validation of an Infarction Code Care Checklist and Determination of its Relationship With Other Patient Safety Indicators: Protocol for a Prospective Study

**DOI:** 10.2196/66584

**Published:** 2025-09-26

**Authors:** Encarna Sanchez Freire, Josep Vidal-Alaball, Aïna Fuster-Casanovas, Queralt Miró Catalina, Joan Cartanyà Bonvehí, Josep Lluis Garcia-Domingo

**Affiliations:** 1 University of Vic-Central University of Catalonia, Faculty of Medicine, Vic Spain; 2 Equip d'Atenció Primària d'Artés, Gerencia Territorial d'Atenció Primària i a la Comunitat de Catalunya Artés Spain; 3 Unitat de suport a la recerca, Gerència d'Atenció Primària i a la Comunitat Catalunya Central, Institut Català de la Salut Sant Fruitos De Bages Spain; 4 eHealth Lab Research Group, School of Health Sciences and eHealth Centrem Universitat Oberta de Catalunya Barcelona Spain; 5 University of Vic-Central University of Catalonia Vic Spain

**Keywords:** myocardial infarction, checklist, patient safety, primary health care, quality indicators, health care

## Abstract

**Background:**

In the care of time-dependent illnesses, facilitating care and systematizing actions with a checklist provides security to health professionals and reduces errors, thereby increasing patient safety. However, despite the widespread use of checklists in other clinical contexts, no studies have yet validated a checklist specifically for infarction code care.

**Objective:**

The objective of this study is to validate the checklist and determine its relationship with the rest of the patient safety indicators in the primary care teams of the Catalan Health Institute of Central Catalonia.

**Methods:**

This is a prospective study for the validation of a checklist for infarction code care. In this study, 2 clinical scenarios of varying difficulty are defined, and the correct answers are established in each case according to the gold standard guidelines. During the first 3 months of the ongoing year, we held an annual training meeting where infarction code referents from various primary care teams gathered to review the new guidelines and outline the training strategy for the next year. These referents conducted annual training sessions for their respective teams before Easter, during which they explained the new guidelines. On the same day as the training, the 2 clinical scenarios were completed using the online version of the checklist for the first time for all participants. The checklist was sent in digital format to all health professionals who responded the first time, and then a reminder was sent to respond a second time at 30, 45, and 90 days to obtain the maximum number of second responses, as the checklist should be completed twice to assess internal reliability and temporal robustness. The number of hits was compared with respect to the gold standard for both the first and the second response. The results obtained from the responses and accuracies, when compared with the gold standards, were evaluated against other available patient safety indicators in the region.

**Results:**

Between January 2023 and May 2023, we obtained 615 responses to the online version of the checklist. We conducted analyses to assess both internal consistency and temporal robustness of the responses and have also structured the framework for comparing these results with other patient safety indicators available in the region. Data analysis is currently underway, and we expect to publish the results in early 2026.

**Conclusions:**

If the checklist demonstrates strong internal consistency and temporal robustness and shows a meaningful relationship with patient safety indicators, it could be implemented across primary care centers using the infarction code. This would support safer, more standardized care in time-sensitive clinical situations.

**Trial Registration:**

IDIAP Jordi Gol 4R22/343; https://idiapjgol.org/grup-recerca/prosaaru/projectes/

**International Registered Report Identifier (IRRID):**

DERR1-10.2196/66584

## Introduction

### Background

Cardiovascular diseases are the leading causes of death worldwide. According to the data from the World Health Organization (WHO), an estimated 17.9 million people died from cardiovascular diseases in 2019, representing 32% of all deaths, of which 85% deaths were from ischemic heart disease and cerebrovascular disease [1].

A cardiovascular risk factor (CVRF) is a biological characteristic of individuals that increases the probability of developing or dying from cardiovascular disease. However, as this is a probability, having no risk factors does not preclude individuals from developing cardiovascular disease nor does the presence of one or more CVRFs necessarily imply a future cardiovascular disease [2]. CVRFs, such as unhealthy diet, physical inactivity, or harmful consumption of alcohol and tobacco, are highly prevalent in Spain. Thus, the optimal setting for a comprehensive approach to the prevention and follow-up of cardiovascular pathologies is primary care.

In the context of primary care, health education through a comprehensive approach to CVRFs is essential in the prevention of cardiovascular diseases. In this regard, the preventive measures to be carried out once cardiovascular risk has been assessed should include interventions aimed at the adoption of healthy lifestyles and relevant pharmacological measures [2]. In addition, the clinical guidelines of the Spanish Society of Cardiology on myocardial infarction state that strategies should focus on minimizing the delay in initiating reperfusion treatment (both by pharmacological and mechanical means) because this pathology is characterized by being time dependent [3].

The latest management guidelines for this pathology establish as a priority an electrocardiogram– percutaneous coronary intervention (PCI) time of no more than 120 minutes [4]. The delay in these times is mainly determined by 3 factors: the delay in the patient’s consultation, the delay in the interpretation of the semiology by the health care professionals, and the delay in transport. Delay in any of these 3 factors leads to an increase in the electrocardiogram-PCI time [5]. Although response times in rural areas are longer than in urban areas, studies suggest that all guidelines should be complied with equally well [4]. Delayed care in rural areas is mainly due to delayed transport, which increases the number of deaths from acute coronary syndrome with ST-segment elevation [5]. Therefore, the potential impact on mortality and morbidity of the measures carried out in the period immediately after acute myocardial infarction (AMI) justifies the development of strategies with the aim of arranging, prioritizing, and systematizing actions. If we implement these strategic measures in scattered population centers that are located farther from the centers where PCI can be performed, the need for such measures becomes even more important.

One possible strategy that could be useful would be to try to minimize the time to initiate treatment. Despite the use of information and communications technologies, experience with training videos for health care personnel is not very evident. However, several sources claim to have evidence of good results for training videos that teach necessary techniques; therefore, greater expertise could reduce the time taken by the practitioner to act [6-8]. The study by Lane et al [9], in which 75 health professionals participated, concluded that training videos improved their knowledge regarding the application of assessment scales. Most studies also show that the comfort level of health professionals with a given technique or procedure increases after viewing a training video [10]. By contrast, designing a checklist and providing training in this tool, so that health professionals are familiar with it, gives security to health professionals and reduces errors in assistance [11,12]. The checklists are applied and validated in surgical care in particular [13,14], and they allow individuals to systematize actions and address them in order of priority, ensuring that if the checklist is followed, adverse incidents in care will be minimized, and no step that compromises the safety of care or patient safety will be forgotten.

Checklists have been studied and implemented in emergency departments not only for the management of specific syndromes but also to systematize actions and guide diagnoses. Their use has been shown to increase adherence to established protocol, reduce differential diagnostic errors, improve patient examination findings in specific syndromes, and decrease errors in test interpretation [15-17]. It has been shown that in situations of cognitive overload, such as emergency departments where long shifts, high-stress, and urgent situations are common, checklists can make diagnosis or interpretation easier as they help manage the overload [18].

Most checklist validations are conducted based on expert consensus, such as the WHO Surgical Safety Checklist [19]; other studies have used Delphi methodology [20,21], and there are no checklist validations published in the literature using the proprietary methodology of questionnaire validation.

In 2017, a checklist for action in the AMI code in Central Catalonia was drawn up with the aim of facilitating data collection and guiding the action to be taken by the primary care health care team. The checklist was formulated by a working group created specifically for this task, along with the participation of primary care physicians from the Catalan Health Institute (ICS) of Central Catalonia, the Territorial Directorate of the Medical Emergency System of Central Catalonia, and the person responsible for the AMI code of the Medical Emergency System of Central Catalonia at the time. A checklist was agreed upon by following the guidelines in force at the time and the care protocols of the 2 institutions.

Central Catalonia is made up of 6 counties: Bages, Berguedà, Lluçanès, Moianès, Osona, and Anoia, corresponding to a population of 525,000 inhabitants and 32 primary care teams (one of which is a prison team). It is mostly rural territory with a high dispersion index. AMI code training is mandatory for all ICS medical and nursing professionals once a year and is done via an online course or through regulated clinical sessions. The sessions are led by the AMI code referents of each team, who are medical or nursing professionals who receive updated training each year and are responsible for disseminating the information from the training to their colleagues. In those training sessions, the use of the checklist is explained to the participants.

The checklist is available in paper format in all emergency departments of primary care centers, local clinics, continuous care points, and primary care emergency centers and also with the prison primary care team. Copies are also available in urgent and emergent care backpacks, as part of our procedures for urgent and emergent situations. However, the use of the checklist is not mandatory, and practitioners can choose whether to use it.

Given this situation of nonuniformity in the use and lack of validation of the tool, it was decided to take advantage of the checklist developed in 2017 and validate it for infarction code (AMI) care in primary care, demonstrating that it can increase patient safety as shown by numerous studies [22-24] or the WHO’s own Surgical Safety Checklist [19].

### Objectives

Therefore, the aim of this study is to validate the infarction code care checklist in terms of internal consistency and temporal robustness and explore its relationship with existing patient safety indicators in primary care settings in Central Catalonia (Table 1).

**Table 1:** Available patient safety indicators.

Indicator	Explanation
GLPI^a^ (incident logging in the territory’s own incident collection application)	Indicator of continuous care notifications; section on urgent care includes incidents involving the care of pathologies that must be treated between primary care and hospital care
EQPF^b^ (pharmaceutical prescription quality standard [25])	Cardiovascular disease indicator and safety self-audit; allows us to know how well or poorly one is prescribing treatment to users with cardiovascular disease
EQA^c^ (standard of care quality [26])	An indicator of cardiovascular disease; reports on the degree of compliance with the agreed-upon care guidelines for cardiovascular diseases and the good or bad monitoring of patients according to the indicators of these guidelines
Number of safety incidents reported to the Safety Incident Notification System of Catalonia [27]	Encompasses the collection of patient safety incidents reported by practitioners related to unscheduled, urgent, and emergent care
Degree of use of the checklists of the Proactive Patient Safety Application of Catalonia [28].	Indicators of the emergency department checklist; indicators of the use and supplementation of the checklist for the equipment and spaces where urgencies and emergencies are treated in primary care centers, which should be used by health professionals

^a^GLPI: incident logging in the territory’s own incident collection application.

^b^EQPF: pharmaceutical prescription quality standard.

^c^EQA: standard of care quality.

## Methods

### Design and Setting of This Study

This is a prospective study for the validation of a checklist for infarction code care. The author of the initial checklist, who is also the leader of the first working group, along with the principal investigator of this study, has updated the 2017 checklist in accordance with the current guidelines (Multimedia Appendix 1). To facilitate data collection during the study, the updated checklist has been converted into a Microsoft 365 Forms questionnaire, an ICS corporate tool, to be completed online.

Two clinical scenarios of different clinical difficulty have been defined. The gold standard was determined by the authors for these 2 scenarios, complementing a checklist for each scenario following the guidelines in force at that time.

To validate the checklist as an effective intervention tool, we compared the information collected in the checklist from 2 proposed clinical scenarios with the gold standard guidelines, asking the health professionals to complement it. This allowed us to determine the internal consistency. In addition, the health professionals were asked to complete the checklist again after a few days. We assessed the temporal robustness by comparing the data collected in the first and second responses.

To ascertain the relationship with patient safety, the available safety indicators (Table 1) allowed us to compare the success of both scenarios with the safety culture of the teams.

### Participants

All physicians and nurses of the 32 primary care teams, the 3 territorial continuous care emergency care teams, and the prison primary care team of Central Catalonia of the ICS who were working from January 2023 to April 2023 were invited to respond, totaling 921 health professionals*.* A total of 921 health professionals were invited to respond.

### Procedure

Starting from the checklist agreed upon in 2017 by a group of experts, the author of the first checklist (from the 2017 proposal) and one of the collaborators and principal investigator of this study updated the paper format with the new European guidelines for action [4].

Once the checklist was updated, it was transformed into an online format via Microsoft 365 Forms to validate it through 2 simulated clinical scenarios (Multimedia Appendix 2) of different difficulty levels and presentations.

The functionality was tested with the authors involved in this study. Once the best option was agreed upon, the checklist was tested with the AMI code referents (n=39) to check its functionality, and they were asked for their opinion and whether they thought improvements were needed to facilitate its implementation. Their inputs were considered, and some changes were made to improve the online version of the checklist.

The correct answers or the correct complementation for both scenarios were defined as the “gold standard.” They were determined for each of the 2 scenarios, complementing a checklist for each scenario following the guidelines in force at that time (Multimedia Appendix 3).

At the same time as the actualization of the checklist, the AMI code referents from the different primary care teams were called to a training meeting to review the criteria for action according to the new guidelines, define how to provide training for the following year, agree on the dates with them to ensure that it has been completed in the first quarter of the current year, and explain them the operation of the checklist.

This procedure is already common in the region; AMI code training is organized through the AMI code referents in each team. They are the ones who receive the updates and pass them on to their teams.

The plan was that the AMI code referents would provide the annual training to the teams, and during that time, they would explain the checklist to the nurses and physicians of their teams and ask them to complete 1 checklist for each clinical scenario. On the same day as the training, the 2 checklists were completed for the first time (day 0). Therefore, the 2 checklists were completed for the first time (day 0) during annual AMI code training by all medical and nursing professionals during the period from January 2023 to May 2023.

**Figure 1:**
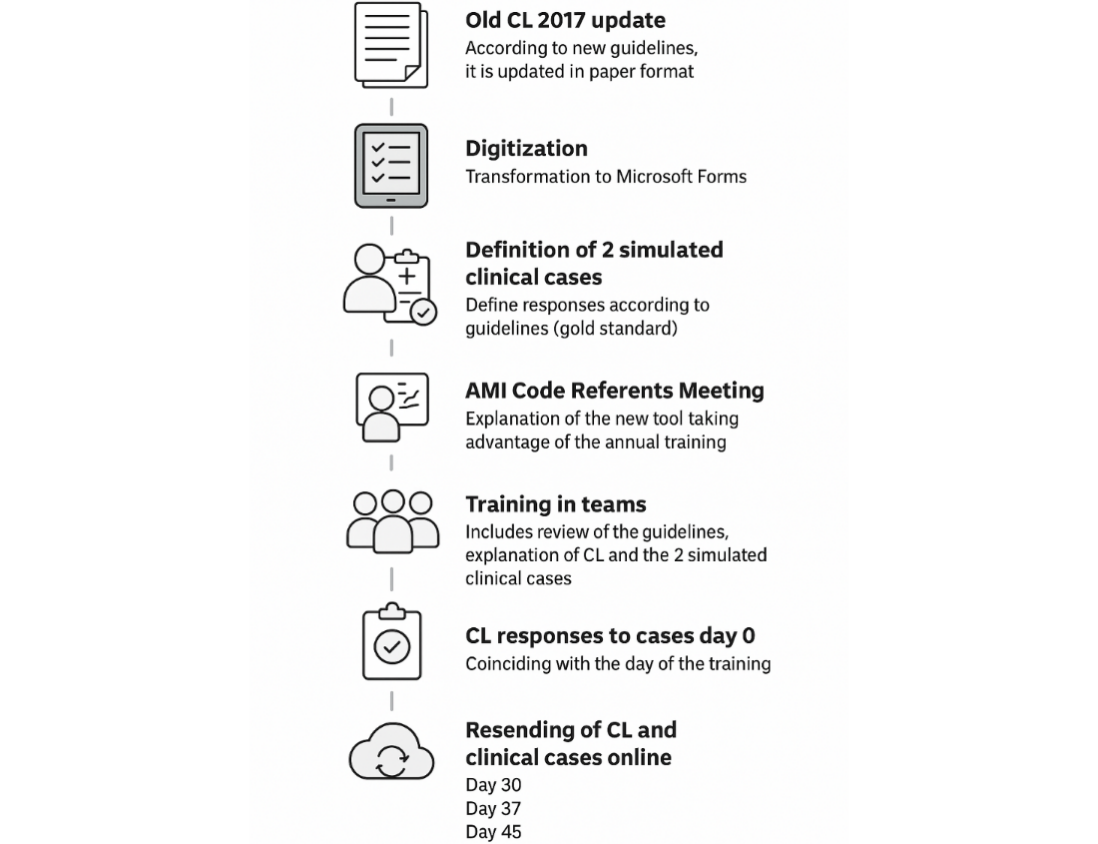
Sampling procedure. AMI: acute myocardial infarction; CL: checklist.

All those health professionals who complete the checklist on day 0 will be asked to complete it again for a second time randomly after 30, 37, or 45 days to see the evolution of the answers over time. Half of them will be sent an email after 30 days, and if they do not answer, an email will be sent again after 37 days. The other half will be emailed after 37 days and, if they do not respond, they will be emailed again after 45 days. There will be a final roundup between 45 and 60 days with a reminder being sent to all those who have not responded (Figure 1).

Potential biases in participant selection could be as follows: health professionals do not attend the annual AMI code training, health professionals do not complete the online version of the checklist during the annual AMI code training, and health professionals do not complete the checklist a second time when it is sent to them online.

The strategies that can possibly avoid these biases are mentioned subsequently. AMI code training is mandatory for all health professionals and is linked to the payment of variable remuneration based on objectives. During the training, AMI code referents supervise and encourage health professionals to participate, and during the second time of completing the checklist, several reminders are sent by email asking participants to complete the checklist.

The questionnaire will have different sections. In the first part of the online questionnaire, health Professionals must read and sign the informed consent and agree to the data protection terms. In this first part, the health professionals will be informed that they need to complete the questionnaire a second time 30, 37, or 45 days after the completion of the first questionnaire, and they will be asked for their corporate email ID (which they can provide voluntarily) so that we can resend the questionnaires.

In the second part of the questionnaire affiliation data will be collected, including health professional category, year of graduation, how many years they have been practicing their profession, how long they have been trained in the AMI code, in which team they work, and the time they need to fill in the checklist. To compare the answers of the same health professional between the first response and the second response to the questionnaire, a code that is the same both times (4 letters of the health card and the last 4 digits of the personal telephone number) must be entered into the questionnaire.

In the third part, the health professionals will then move on to the questionnaire part, where they will find the clinical scenarios and then the questions or actions of the checklist that they will have to check or complete. Only completed checklists will be analyzed. To proceed with the online version, selecting an option is mandatory; questionnaires that are not completed till the end will not be counted, as incomplete responses are not saved in Microsoft Forms 365.

### Sampling Method

This study followed a convenience sampling method. A total of 921 medical and nursing professionals were invited to participate, and the questionnaire was sent out at 30, 45, and 90 days to respond again.

To obtain estimates with 95% CIs and a replacement rate of 5% and assuming percentages of 50% to ensure all estimates, 405 health professionals will need to respond to the questionnaire.

### Statistical Analysis

The sociodemographic variables of the sample of health professionals who responded to the checklist will be described by means of absolute frequencies and percentages. To evaluate the use of the checklist of clinical scenarios, a total score will be obtained for each case by adding the total number of items answered correctly. The total score for clinical scenario 1 is 40 points, and the total score for clinical scenario 2 is 42 points. There are 2 questions in the 2 scenarios where the maximum score is 2: name and onset of pain. In these 2 questions, if answered correctly, a score of 2 points is obtained; conversely, if answered with minor variations in the correct answer, a score of 1 point is obtained. The scoring system is accurately explained in Multimedia Appendix 3.

The results were converted to a 10-point scale for easier interpretation. The total scores of each case were described for the total sample and according to each category of the variables analyzed with means and SDs. ANOVA contrast will be used to compare scores between categories. In addition, the percentage of items answered correctly or incorrectly will be evaluated.

To validate the checklist, the “Fleiss κ” value will be estimated to analyze internal consistency and the intraclass correlation coefficient value to evaluate temporal consistency. In addition, the scores obtained in each clinical scenario will be compared between the response times (test retest).

The analyses will be performed using the statistical software R (version 4.2.1; R Foundation for Statistical Computing); the 95% CIs and the significance level will be set at 5%. On the one hand, we will have the responses collected from the checklist questionnaire sent using Microsoft 365 Forms, and on the other hand, we will have the data from the dashboards of the different patient safety applications (Table 1).

### Ethical Considerations

All data extracted for the study will be anonymous, and confidentially will be maintained following the ethical principles of the 1964 Declaration of Helsinki, the European Data Protection Regulation 2016/679, and the Spanish legislation via Law 3/2018 of April 27 on Data Protection. The Organic Law 3/2018 of December 5 on the Protection of Personal Data and the Guarantee of Digital Rights will also be complied with.

We ask for consent in the first part of the online questionnaire as stated in the Procedure section. All information will be treated confidentially; there is a personal commitment to confidentiality that prevents the dissemination of the data consulted for the preparation of the work.

The research team will have no access to identifying data of the participants, directly or indirectly, under any circumstances. The participants will be informed in the same online questionnaire about the objectives of the study and will also be asked to accept the conditions by signing the informed consent in the same questionnaire. The participants did not receive any financial or material compensation for their participation in the study.

To validate the checklist, the data from the checklist questionnaire database will be coded because the participants who complete the checklist will generate a code, which the researchers can then cross-check to compare results, and at no time will they be able to reidentify the person.

This study received ethics approval from the research ethics committee of the Foundation University Institute for Primary Health Care Research Jordi Gol i Gurina (22/211-P).

## Results

We collected 615 responses to the online version of the checklist between January 2023 and May 2023. Throughout 2024, we analyzed the internal consistency and temporal robustness of the responses. The framework for integrating these results with patient safety indicators from various applications has been developed, although the comparative analysis is not yet included in this version of the manuscript. Data analysis is currently underway, and we expect to publish the results in early 2026.

## Discussion

### Overview

This study aims to validate a checklist designed for infarction code care in primary care settings by assessing its internal consistency and temporal robustness. We anticipate that the checklist will demonstrate strong reliability across repeated applications and that higher checklist scores will be associated with better performance on established patient safety indicators. These findings would support the checklist’s potential as a useful tool for standardizing care and enhancing patient safety in a time-dependent clinical situation as AMI.

### Anticipated Findings

The primary objective of developing and validating a checklist is to enhance patient safety. The use of simulated clinical cases or scenarios has been used by various authors as a method for testing checklists [11,24]. Therefore, the proposed study represents a valuable approach to validation, particularly given the limited number of existing studies that go beyond expert consensus or the Delphi methodology [20,21].

### Comparison to Prior Work

According to Kohn et al [29], checklists play a crucial role in the patient safety improvement process. First, they help avoid reliance on the health professional’s memory. By standardizing processes and reducing dependence on the memory of health professionals who may be unfamiliar with the procedures or equipment, checklists contribute to safer care. Both protocols and checklists in this context can enhance patient safety. Second, checklists help reduce the need for health professionals to remain vigilant for extended periods. By providing a checklist and mandating its use, some actions can be automated, further improving safety.

However, checklists can also increase patient safety indirectly by improving other aspects of care. They increase risk identification and decrease errors [24]; improve emergency management and guide the actions of health professionals [11]; provide a record of tasks or actions to be performed, increasing the likelihood that they will be performed [30]; increase cooperation among health professionals; and prevent some adverse effects [22].

It has also been shown that the identification of communication errors is the common root cause of many adverse events and patient safety incidents. This fact has motivated the development of actions to improve communication, including the development of checklists that reduce the variability of clinical practice, standardize the transmission of information, and help avoid forgetting some details [31].

### Strengths and Limitations

In AMI code care, especially in primary care and in rural areas where extensive travel is required, the availability of a checklist may be essential to guide the actions of nonexpert professionals [11] in a pathology that is time dependent [4]. Working with checklists provides a record of the work to be done, but it is difficult to know if it has actually been done as stipulated. However, the mere reminder of the things to do from the checklist increases the safety of the patient, no matter how little the increment is [30].

That being said, it should be kept in mind that checklists only work if there is a knowledge base to organize clinical practice, and they are not a “one size fits all” solution. The systematization of compliance of “done” tasks is not a guarantee that they are really done or that other details not contemplated in the checklist are not missing [31], and training for their use is needed [24].

Although checklists are not a definitive solution, they can standardize care by decreasing variability and complications and allowing for continuous quality improvement of care teams [11]. In this sense, the availability of a validated, contrasted tool that is adjusted to the knowledge of the health professionals of the territory can guide the actions and improve the care of patients with this pathology.

The main limitations of the study are that the response time of the checklist is long and that it has to be answered twice, which can lead to participant losses, considering that the maximum number of people who can respond is approximately 900. Furthermore, the degree of training in the AMI code is different among health professionals. Although training is mandatory for health professionals once a year, it cannot be assured that they have completed it or that they have completed other complementary training. In addition, care guidelines are frequently updated, and we may encounter changes in the gold standard. Another limitation is the problem of external validation due to a small convenience sample without geographic stratification or stratification according to health professionals’ experiences as well as the ecological validity of the study due to using only simulated instances and not real cases.

As for the last limitation, we might find that health professionals do not agree to being contacted via corporate email after 30, 37, or 45 days.

### Future Directions

The analysis of checklist responses and their persistence over time (at 30, 37, and 45 days) will provide insight into whether health professionals retain the necessary knowledge and are able to act appropriately following training. Furthermore, it will help identify potential deficiencies in training or management, as reflected by unsatisfactory responses. Teams that perform well in quality and prescription standards typically demonstrate a strong patient safety culture and, consequently, are expected to achieve higher scores in the completion of other checklists (Proactive Patient Safety Application of Catalonia) [28] and in patient safety applications, such as the Safety Incident Notification System of Catalonia [27].

In parallel, the checklist could be further validated with more health professionals to have a more representative sample and ensure external validity as well as incorporate real-world cases in addition to the simulated ones.

### Dissemination Plan

If the checklist is validated, we plan to disseminate it across all primary care teams of the ICS in Central Catalonia and the rest of the ICS teams. The dissemination strategy will include the development of training materials, integration into annual AMI code training sessions, and publication of implementation guidelines through institutional channels. Results will be shared in professional meetings and presentations. In addition, we aim to publish the final findings in peer-reviewed journals and present them at national and international conferences to support broader adoption and encourage replication in other health care settings.

## Appendix

Multimedia Appendix 1 Checklist description.

Multimedia Appendix 2 Clinical scenario description.

Multimedia Appendix 3 Gold standard responses.

## Abbreviations

AMI: acute myocardial infarction
CVRF: cardiovascular risk factor
ICS: Catalan Health Institute
PCI: percutaneous coronary intervention
WHO: World Health Organization

## Data Availability

The datasets generated or analyzed during this study are not publicly available but are available from the corresponding author on reasonable request.

## References

[1] (2023). Cardiovascular diseases (CVDs). World Health Organization.

[2] Lobos Bejarano JM, Brotons Cuixart C (2011). Factores de riesgo cardiovascular y atención primaria: evaluación e intervención. Aten Primaria.

[3] Arós F, Loma-Osorio Á, Alonso Á, Alonso JJ, Cabadés A, Coma-Canella I, García-Castrillo L, García E, López de Sá E, Pabón P, San José JM, Vera A, Worner F (1999). Guías de actuación clínica de la Sociedad Española de Cardiología en el infarto agudo de miocardio. Rev Esp Cardiol.

[4] Ibáñez B, James S, Agewall S, Antunes MJ, Bucciarelli-Ducci C, Bueno H, Caforio AL, Crea F, Goudevenos JA, Halvorsen S, Hindricks G, Kastrati A, Lenzen MJ, Prescott E, Roffi M, Valgimigli M, Varenhorst C, Vranckx P, Widimsk P, Collet J, Kristensen SD, Aboyans V, Baumbach A, Bugiardini R, Mircea Coman I, Delgado V, Fitzsimons D, Gaemperli O, Gershlick AH, Gielen S, Harjola V, Katus HA, Knuuti J, Kolh P, Leclercq C, Lip GY, Morais J, Neskovic AN, Neumann F, Niessner A, Piepoli MF, Richter DJ, Shlyakhto E, Simpson IA, Steg G, Terkelsen CJ, Thygesen K, Windecker S, Zamorano JL, Zeymer U (2017). Guía ESC 2017 sobre el tratamiento del infarto agudo de miocardio en pacientes con elevación del segmento ST. Rev Esp Cardiol.

[5] Beig JR, Tramboo NA, Kumar K, Yaqoob I, Hafeez I, Rather FA, Shah TR, Rather HA (2017). Components and determinants of therapeutic delay in patients with acute ST-elevation myocardial infarction: a tertiary care hospital-based study. J Saudi Heart Assoc.

[6] Pan M, Harcharik S, Moskalenko M, Luber A, Bernardo S, Levitt J (2014). Instructional video for teaching venepuncture. Clin Teach.

[7] Levitan RM, Goldman TS, Bryan DA, Shofer F, Herlich A (2001). Training with video imaging improves the initial intubation success rates of paramedic trainees in an operating room setting. Ann Emerg Med.

[8] Nousiainen M, Brydges R, Backstein D, Dubrowski A (2008). Comparison of expert instruction and computer-based video training in teaching fundamental surgical skills to medical students. Surgery.

[9] Lane PL, Báez AA, Brabson T, Burmeister DD, Kelly JJ (2002). Effectiveness of a Glasgow coma scale instructional video for EMS providers. Prehosp Disaster Med.

[10] Srivastava G, Roddy M, Langsam D, Agrawal D (2012). An educational video improves technique in performance of pediatric lumbar punctures. Pediatr Emerg Care.

[11] Subbe CP, Kellett J, Barach P, Chaloner C, Cleaver H, Cooksley T, Korsten E, Croke E, Davis E, De Bie AJ, Durham L, Hancock C, Hartin J, Savijn T, Welch J, Crisis Checklist Collaborative (2017). Crisis checklists for in-hospital emergencies: expert consensus, simulation testing and recommendations for a template determined by a multi-institutional and multi-disciplinary learning collaborative. BMC Health Serv Res.

[12] Norton EK, Singer SJ, Sparks W, Ozonoff A, Baxter J, Rangel S (2016). Operating room clinicians' attitudes and perceptions of a pediatric surgical safety checklist at 1 institution. J Patient Saf.

[13] Sparkes D, Rylah B (2010). The World Health Organization surgical safety checklist. Br J Hosp Med (Lond).

[14] Dabholkar Y, Velankar H, Suryanarayan S, Dabholkar TY, Saberwal AA, Verma B (2018). Evaluation and customization of WHO safety checklist for patient safety in otorhinolaryngology. Indian J Otolaryngol Head Neck Surg.

[15] Ko HC, Turner TJ, Finnigan MA (2011). Systematic review of safety checklists for use by medical care teams in acute hospital settings--limited evidence of effectiveness. BMC Health Serv Res.

[16] Ely JW, Graber ML, Croskerry P (2011). Checklists to reduce diagnostic errors. Acad Med.

[17] Graber ML, Sorensen AV, Biswas J, Modi V, Wackett A, Johnson S, Lenfestey N, Meyer AN, Singh H (2014). Developing checklists to prevent diagnostic error in emergency room settings. Diagnosis (Berl).

[18] Sibbald M, de Bruin AB, van Merrienboer JJ (2013). Checklists improve experts' diagnostic decisions. Med Educ.

[19] (2009). WHO guidelines for safe surgery 2009: safe surgery saves lives. World Health Organization.

[20] Tsai HH, Tsai YF (2018). Development, validation and testing of a nursing home to emergency room transfer checklist. J Clin Nurs.

[21] Desnoyer A, Blanc AL, Pourcher V, Besson M, Fonzo-Christe C, Desmeules J, Perrier A, Bonnabry P, Samer C, Guignard B (2017). PIM-Check: development of an international prescription-screening checklist designed by a Delphi method for internal medicine patients. BMJ Open.

[22] Askarian M, Kouchak F, Palenik CJ (2011). Effect of surgical safety checklists on postoperative morbidity and mortality rates, Shiraz, Faghihy Hospital, a 1-year study. Qual Manag Health Care.

[23] Oak SN, Dave NM, Garasia MB, Parelkar SV (2015). Surgical checklist application and its impact on patient safety in pediatric surgery. J Postgrad Med.

[24] Scott SS, Henneman EA, Nathanson BH, Andrzejewski C, Gonzalez M, Walker R, Martinez VI (2020). Use of a transfusion checklist by student nurses to improve patient safety. J Nurses Prof Dev.

[25] Indicadors de l'atenció primària. Gencat.

[26] Equitat. Gencat.

[27] (2023). Seguretat dels Pacients. Gencat.

[28] (2023). Aplicació proactiva de geguretat dels pacients de Catalunya (PROSP Cat). Gencat.

[29] Kohn LT, Corrigan JM, Donaldson MS (2000). To Err is Human: Building a Safer Health System.

[30] Papoutsi C, Poots A, Clements J, Wyrko Z, Offord N, Reed J (2018). Improving patient safety for older people in acute admissions: implementation of the Frailsafe checklist in 12 hospitals across the UK. Age Ageing.

[31] Hilligoss B, Moffatt-Bruce SD (2014). The limits of checklists: handoff and narrative thinking. BMJ Qual Saf.

