# Supplementary material for: Validation of an Infarction Code Care Checklist and Determination of its Relationship With Other Patient Safety Indicators: Protocol for a Prospective Study
Source: JMIR Res Protoc. 2025 Sep 26;14:e66584. doi: 10.2196/66584 (PMC12514402; doi:10.2196/66584)
Supplement: Multimedia Appendix 1 [file resprot_v14i1e66584_app1.docx]

**Appendix 1:**

**Description of infarct code checklist without responses, the same structure that health professionals can see in on-line version.**

**Affiliation:**

- Name
- Personal Identification Code (CIP)
- Age (in years)
- Sex
- Weight (Kg)
- Date

**Chronology:**

- Pain onset time
- Consultation time
- Time of ECG
- Time of transfer to SEM

**AMI code activation criteria:** chest pain or symptoms suggestive of acute coronary syndrome ≥ 30 min (with persistent changes within 5 min of administering Nitro-glycerine (NTG)).

1. New ST-segment elevation at the J point in 2 contiguous leads:
   1. ≥1mm to any lead, except V1-V3.
   2. V1-V3 leads:
      1. ≥ 2mm in males > 40 years.
      2. ≥ 2.5mm in males < 40 years.
      3. ≥ 1.5mm in females, regardless of age.
   3. Newly emerging LBBB
   4. Newly emerging RBBB
2. ST segment depression ≥ 1mm in 8 or more leads + ST elevation to aVR.
3. Isolated ST-segment depression ≥ 0.5mm in leads V1-V3 / ST-segment elevation in posterior leads (V7-V9).
4. Pacemaker rhythm (During RV pacing, the ECG also shows RBBB, and the above criteria are also applicable but are less specific).

**Activate AMI code**:

- Done
- Time

**Monitoring and AED nearby**

- Done

**Pathological history:**

- CVRF
  - AHT
  - DM
  - Dyslipidaemia (DL)
  - Smoking
- Ischemic heart disease
- Do they take anticoagulant treatment? (OAC or NOACs)
  - Yes
  - No
- ASA allergy?
  - Yes
  - No
- Allergies to drugs or iodinated contrast agents?
  - No
  - Yes
    - Which ones?

**Physical examination**

- - Blood pressure (mmHg)
  - Heart rate (beats per min)
  - Respiratory rate (breaths per min)
  - Oxygen saturation (%)
  - Glycemia (correct with rapid insulin if Glycemia > 200mg/dl) mg/dl
  - Killip
    - I: Normal RS, no JVD or AJR
    - II: wet crackles + JVD or AJR
    - III: acute pulmonary oedema
    - IV: cardiogenic shock

**Venous access to LUL** (avoid wrist)

- Done
- Size (G)

**Treatment:**

1. Aspirin 250mg always if no allergy. If the patient is not vomiting, then it must be taken orally:
   - Done
   - Time
2. Nitro-glycerine 0.4mg SL if pain and SBP>90 mmHg, HR>50x' and no suspicion of RV AMI:
   - Done
   - Dosage
3. If pain persists:
   1. Morphic Chloride IV (dilute 10mg Morphic Chloride in 9ml SF)
   - Done
   - Dosage (2-3mg every 5min, maximum dose 10mg)
   1. Or Fentanyl IV (undiluted)
   - Done
   - Dosage (50mcg (1cc) every 5 min, maximum 150mcg (3cc))
4. Oxygen only if O2 saturation <90% (To achieve O2 saturations ≥90%)
   - Done
   - It is not necessary
5. Other treatments: Diazepam, Primperan or Insulin

**Repeat ECG** 5 min after initiating nitrite treatment or after pain subsides

- Done
- Does ST alteration persist?
  - Yes
  - No

**Resolution**

AMI code confirmed?

- - Yes
  - No
- Transfer for angioplasty?
  - Yes
  - No
- Fibrinolysis
  - Yes
  - No
- Cardiorespiratory arrest?
  - No
  - Yes
  - CRA recovered
