# Supplementary material for: Validation of an Infarction Code Care Checklist and Determination of its Relationship With Other Patient Safety Indicators: Protocol for a Prospective Study
Source: JMIR Res Protoc. 2025 Sep 26;14:e66584. doi: 10.2196/66584 (PMC12514402; doi:10.2196/66584)
Supplement: Multimedia Appendix 2 [file resprot_v14i1e66584_app2.docx]

**Appendix 2:**

**Description of the clinical scenarios**

CLINICAL CASE 1: Low difficulty

Joan Farre Tort

52-year-old man with Personal Identification Code (CIP) FATO0700522001

With pathological history of diabetes, hypertension, and dyslipidaemia. Weighs 102 kg. He has no known drug allergies or other history.

Consultation on 5th April 2020 at the Massaners primary care centre at 17:00.

He reported chest tightness as a "weight in the chest", shortness of breath, profuse sweating, and nausea during about 15 minutes.

An electrocardiogram (ECG) was performed at 10 minutes showing ST elevation greater than 2mm from V2 to V4 (we do not show the ECG image because the aim of the study is not to measure ECG reading skills).

At 17:15, the AMI code was activated, the patient was fitted with the monitor and the defibrillator was retrieved. The patient's vital signs were taken, and the patient was examined. BP: 180/90, HR: 100 x', RR: 18 x', Sat.O2: 98%, glycemia 180 mg/dl. Upon cardiorespiratory examination, he presented with wet bibasilar crackles up to midline position, rhythmic heart sounds without murmurs, no distal oedema, or signs of abdominojugular reflux.

21 G venous access was placed.

After 30 minutes of arrival, 250 mg of aspirin, 0.8 mg of Solinitrina SL and 2 cc of diluted morphic chloride (1/9) were administered. An ECG was performed and showed no changes with respect to the previous one.

The AMI code was confirmed on arrival of advanced life support from SEM at 17:40, and they were transferred to hemodynamics for angioplasty, which confirmed occlusion.

CLINICAL CASE 2: High difficulty

Julia Cases Pla

60-year-old woman with Personal Identification Code (CIP) CAPL1581025001

Consultation on 8th October 2020 at the Tèrmens Primary Care Centre at 12 p.m.

She has been accompanied by the owner of the store next door to the Primary Care Centre because she says she is unable to speak. She says that she has been feeling strange for a few days: she has choking and malaise with epigastric discomfort that she does not know how to explain better. While in the store next door to the Primary Care Centre she became dizzy, and the chest discomfort became more intense.

She reports no drug allergies or history of interest, but she wears a nitrate patch and says she sometimes has elevated blood pressure but does not need medication. She also reports that she cannot take nonsteroidal anti-inflammatory drugs because the last time she took an aspirin she got red spots on her skin that itched and made it hard to breathe. We do not have her records because she is away on holiday alone. She believes she weighs about 70 kg.

An electrocardiogram (ECG) was performed at 10 minutes showing an ST elevation of 2mm at DIII and 1mm at aVF (we do not show the ECG image because the aim of the study is not to measure ECG reading skills).

At 12:20, the AMI code was activated, the monitor was placed on the patient and the defibrillator was retrieved. The patient's vitals were taken, and the patient was examined BP: 85/60, HR: 50 x', RR: 18 x', Sat.O2: 92%, glycemia 80 mg/dl. Upon cardiorespiratory examination she presented with bibasilar wet crackles, rhythmic heart sounds without murmurs and jugular engorgement, distal oedema with fovea.

20G venous access was placed. After 30 minutes of her arrival, an ECG was performed and showed no changes with respect to the previous one.

As she was very restless, we administered Diazepam 5mg SL.

The AMI code was confirmed with the arrival of SEM advanced life support at 13:00 and she was transferred to hemodynamic for angioplasty, which confirmed occlusion.
