# Supplementary material for: Validation of an Infarction Code Care Checklist and Determination of its Relationship With Other Patient Safety Indicators: Protocol for a Prospective Study
Source: JMIR Res Protoc. 2025 Sep 26;14:e66584. doi: 10.2196/66584 (PMC12514402; doi:10.2196/66584)
Supplement: Multimedia Appendix 3 [file resprot_v14i1e66584_app3.docx]

**Appendix 3:**

**Responses expected in the complementation of the checklist according to guidelines an assumed as a gold standard.**

**The answers have been answered alongside and the required actions marked in bold. If there is no answer or nothing marked in bold it is because nothing specific needed to be done according to the description of clinical scenario and current guidelines.**

**This have been doing for the 2 clinical scenarios.**

**CLINICAL SCENARIO 1:**

**Affiliation:**

- Name: Joan Farre Tort (The correct name for 2 points is Joan Farre Tort and the variation of only one surname has been considered correct with 1 point - Joan Farre).
- Personal Identification Code (CIP): FATO0700522001
- Age (in years): 52
- Sex: male
- Weight (Kg): 102
- Date: 05/04/2020

**Chronology:**

- Pain onset time: 16:45 (The correct starting time of the pain for 2 points was 16:45, but 1 point was given to those who said between 16:35 and 16:55).
- Consultation time: 17:00
- Time of ECG: 17:10
- Time of transfer to SEM: 17:40

**AMI code activation criteria:** chest pain or symptoms suggestive of acute coronary syndrome ≥ 30 min (with persistent changes within 5 min of administering Nitro-glycerine (NTG)).

1. **New ST-segment elevation at the J point in 2 contiguous leads:**
   1. ≥1mm to any lead, except V1-V3.
   2. V1-V3 leads:
      1. ≥ 2mm in males > 40 years.
      2. ≥ 2.5mm in males < 40 years.
      3. ≥ 1.5mm in females, regardless of age.
   3. Newly emerging LBBB
   4. Newly emerging RBBB
2. ST segment depression ≥ 1mm in 8 or more leads + ST elevation to aVR.
3. Isolated ST-segment depression ≥ 0.5mm in leads V1-V3 / ST-segment elevation in posterior leads (V7-V9).
4. Pacemaker rhythm (During RV pacing, the ECG also shows RBBB, and the above criteria are also applicable but are less specific).

**Activate AMI code**:

- Done: yes
- Time: 17:15

**Monitoring and AED nearby**

- Done: yes

**Pathological history:**

- CVRF
  - AHT: Yes
  - DM: Yes
  - Dyslipidaemia (DL): Yes
  - Smoking: No
- Ischemic heart disease: No
- Do they take anticoagulant treatment? (OAC or NOACs)
  - Yes
  - **No**
- ASA allergy?
  - Yes
  - **No**
- Allergies to drugs or iodinated contrast agents?
  - **No**
  - Yes
    - Which ones?

**Physical examination**

- - Blood pressure (mmHg): 180/90
  - Heart rate (beats per min): 100
  - Respiratory frequency (breaths per min): 18
  - Oxygen saturation (%): 98
  - Glycemia (correct with rapid insulin if Glycemia > 200mg/dl) mg/dl: 180
  - Killip
    - I: Normal RS, no JVD or AJR
    - **II: wet crackles + JVD or AJR**
    - III: acute pulmonary oedema
    - IV: cardiogenic shock

**Venous access to LUL** (avoid wrist)

- Done: yes
- Size (G): 21

**Treatment:**

1. Aspirin 250mg always if no allergy. If the patient is not vomiting, then it must be taken orally:
   - Done: yes
   - Time: 17:30
2. Nitro-glycerine 0.4mg SL if pain and SBP>90 mmHg, HR>50x' and no suspicion of RV AMI:
   - Done: yes
   - Dosage: 2 puffs or 1 tablet
3. If pain persists:
   1. **Morphic Chloride IV** (dilute 10mg Morphic Chloride in 9ml SF)
   - Done: yes
   - Dosage (2-3mg every 5min, maximum dose 10mg): 2mg
   1. Or Fentanyl IV (undiluted)
   - Done
   - Dosage (50mcg (1cc) every 5 min, maximum 150mcg (3cc))
4. Oxygen only if O2 saturation <90% (To achieve O2 saturations ≥90%)
   - Done
   - **It is not necessary**
5. Other treatments: Diazepam, Primperan or Insulin. No

**Repeat ECG** 5 min after initiating nitrite treatment or after pain subsides

- Done: yes
- Does ST alteration persist?
  - **Yes**
  - No

**Resolution**

- AMI code confirmed?
  - **Yes**
  - No
- Transfer for angioplasty?
  - **Yes**
  - No
- Fibrinolysis
  - Yes
  - **No**
- Cardiorespiratory arrest?
  - **No**
  - Yes
  - CRA recovered

**CLINICAL SCENARIO 2:**

**Affiliation:**

- Name: Julia Cases Pla (The correct name for 2 points is Julia Cases Pla and the variation of only one surname has been considered correct with 1 point - Julia Cases).
- Personal Identification Code (CIP): CAPL1581025001
- Age (in years): 60
- Sex: female
- Weight (Kg): 70
- Date: 08/10/2020

**Chronology:**

- Pain onset time: 11:50 (The correct starting time of the pain for 2 points was 11:50, but 1 point was given to those who said between 11:40 and 12:00).
- Consultation time: 12:00
- Time of ECG: 12:10
- Time of transfer to SEM: 13:00

**AMI code activation criteria:** chest pain or symptoms suggestive of acute coronary syndrome ≥ 30 min (with persistent changes within 5 min of administering Nitro-glycerine (NTG)).

1. **New ST-segment elevation at the J point in 2 contiguous leads:**
   1. ≥1mm to any lead, except V1-V3.
   2. V1-V3 leads:
      1. ≥ 2mm in males > 40 years.
      2. ≥ 2.5mm in males < 40 years.
      3. ≥ 1.5mm in females, regardless of age.
   3. Newly emerging LBBB
   4. Newly emerging RBBB
2. ST segment depression ≥ 1mm in 8 or more leads + ST elevation to aVR.
3. Isolated ST-segment depression ≥ 0.5mm in leads V1-V3 / ST-segment elevation in posterior leads (V7-V9).
4. Pacemaker rhythm (During RV pacing, the ECG also shows RBBB, and the above criteria are also applicable but are less specific).

**Activate AMI code**:

- Done: yes
- Time: 12:20

**Monitoring and AED nearby**

- Done: yes

**Pathological history:**

- CVRF
  - AHT: yes
  - DM: no
  - Dyslipidaemia (DL): no
  - Smoking: no
- Ischemic heart disease: yes
- Do they take anticoagulant treatment? (OAC or NOACs)
  - Yes
  - **No**
- ASA allergy?
  - **Yes**
  - No
- Allergies to drugs or iodinated contrast agents?
  - **No**
  - Yes
    - Which ones?

**Physical examination**

- - Blood pressure (mmHg): 85/60
  - Heart rate (beats per min): 50
  - Respiratory frequency (breaths per min): 18
  - Oxygen saturation (%): 92
  - Glycemia (correct with rapid insulin if Glycemia > 200mg/dl) mg/dl: 80
  - Killip
    - I: Normal RS, no JVD or AJR
    - II: wet crackles + JVD or AJR
    - **III: acute pulmonary oedema**
    - IV: cardiogenic shock

**Venous access to LUL** (avoid wrist)

- Done: yes
- Size (G): 20

**Treatment:**

1. Aspirin 250mg always if no allergy. If the patient is not vomiting, then it must be taken orally:
   - Done: no
   - Time
2. Nitro-glycerine 0.4mg SL if pain and SBP>90 mmHg, HR>50x' and no suspicion of RV AMI:
   - Done: no
   - Dosage
3. If pain persists:
   1. Morphic Chloride IV (dilute 10mg Morphic Chloride in 9ml SF)
   - Done: no
   - Dosage (2-3mg every 5min, maximum dose 10mg)
   1. Or Fentanyl IV (undiluted)
   - Done: no
   - Dosage (50mcg (1cc) every 5 min, maximum 150mcg (3cc))
4. Oxygen only if O2 saturation <90% (To achieve O2 saturations ≥90%)
   - Done
   - **It is not necessary**
5. Other treatments: Diazepam, Primperan or Insulin: Diazepam 5mg SL

**Repeat ECG** 5 min after initiating nitrite treatment or after pain subsides

- Done: yes
- Does ST alteration persist?
  - **Yes**
  - No

**Resolution**

- AMI code confirmed?
  - **Yes**
  - No
- Transfer for angioplasty?
  - **Yes**
  - No
- Fibrinolysis
  - Yes
  - **No**
- Cardiorespiratory arrest?
  - **No**
  - Yes
  - CRA recovered
